# Antisense Oligonucleotide-Mediated Splice Switching: Potential Therapeutic Approach for Cancer Mitigation

**DOI:** 10.3390/cancers13215555

**Published:** 2021-11-05

**Authors:** Prithi Raguraman, Akilandeswari Ashwini Balachandran, Suxiang Chen, Sarah D. Diermeier, Rakesh N. Veedu

**Affiliations:** 1 Centre for Molecular Medicine and Innovative Therapeutics, Murdoch University, Murdoch, WA 6150, Australia; Prithi.Raguraman@murdoch.edu.au (P.R.); Akila.Balachandran@murdoch.edu.au (A.A.B.); S.Chen@murdoch.edu.au (S.C.); 2 Perron Institute for Neurological and Translational Science, Nedlands, WA 6009, Australia; 3 Department of Biochemistry, University of Otago, Dunedin 9016, New Zealand; sarah.diermeier@otago.ac.nz

**Keywords:** antisense oligonucleotide, splice switching, cancer therapy

## Abstract

**Simple Summary:**

Splicing is an important mechanism by which precursor mRNA is modified into mature mRNA. This splicing plays a major role in the generation of different proteins required for cells. Cancer cells modulate this splicing in such a way that it facilitates uncontrolled growth and survival. Cancer is one of the leading causes of death, and the therapies that are currently available also affect normal cells. Antisense oligonucleotides (AOs) are synthetic DNA/RNA that bind specifically to target mRNA and thereby have fewer off-target effects. These AOs have the potential to modulate the splicing mechanism. In this review, we will discuss the different modes of action of AOs and their potential in targeting cancer.

**Abstract:**

Splicing is an essential process wherein precursor messenger RNA (pre-mRNA) is reshaped into mature mRNA. In alternative splicing, exons of any pre-mRNA get rearranged to form mRNA variants and subsequently protein isoforms, which are distinct both by structure and function. On the other hand, aberrant splicing is the cause of many disorders, including cancer. In the past few decades, developments in the understanding of the underlying biological basis for cancer progression and therapeutic resistance have identified many oncogenes as well as carcinogenic splice variants of essential genes. These transcripts are involved in various cellular processes, such as apoptosis, cell signaling and proliferation. Strategies to inhibit these carcinogenic isoforms at the mRNA level are promising. Antisense oligonucleotides (AOs) have been developed to inhibit the production of alternatively spliced carcinogenic isoforms through splice modulation or mRNA degradation. AOs can also be used to induce splice switching, where the expression of an oncogenic protein can be inhibited by the induction of a premature stop codon. In general, AOs are modified chemically to increase their stability and binding affinity. One of the major concerns with AOs is efficient delivery. Strategies for the delivery of AOs are constantly being evolved to facilitate the entry of AOs into cells. In this review, the different chemical modifications employed and delivery strategies applied are discussed. In addition to that various AOs in clinical trials and their efficacy are discussed herein with a focus on six distinct studies that use AO-mediated exon skipping as a therapeutic strategy to combat cancer.

## 1. Introduction

RNA splicing is a form of RNA processing in which a freshly made precursor messenger RNA (pre-mRNA, or primary transcript) is transformed into a mature mRNA. The process of splicing is catalyzed by the RNA-protein complex known as the spliceosome. The spliceosome consists of five small nuclear ribonucleoproteins (snRNPs), U1, U2, U4, U5 and U6 [1]. During splicing, introns are removed, and exons are joined together.

Splicing can create a range of transcript variants by varying the exon composition of the same transcript. This phenomenon is termed alternative splicing. Alternative splicing is an indispensable process for expanding the spatiotemporal complexity of the transcriptome [2]. More specifically, it is the process wherein the exons of any primary transcript get rearranged to form an mRNA splice variant, and subsequently, the protein isoform, which are distinct both structurally and functionally [3]. In humans, more than 70% of multiexon genes undergo alternative splicing [4]. These splicing events are very tightly regulated and are highly tissue-specific [5]. Different cis- and trans-acting factors regulate the splicing process as they determine the splice site selection [6]. These splice sites can be weakened or strengthened due to point mutations, ultimately changing the splicing events, resulting in disease [6]. Furthermore, in most eukaryotic organisms, there are certain sequences that are ignored by the splicing machinery. These are known as the pseudoexons. In recent years, it has been reported that atypical inclusion of pseudoexons is more frequent than previously understood and is implicated in many human diseases, such as Duchenne muscular dystrophy (DMD) [7].

Cancer is caused by abnormal cell proliferation resulting from altered gene expression [8]. This can include overexpression of oncogenes [9] and production of aberrant splice variants, which subsequently leads to synthesis of carcinogenic protein isoforms [10]. In the past few decades, new developments into understanding the biological basis for cancer progression and therapeutic resistance have led to the identification of many alternative splicing events, which are key in the development of cancer. These carcinogenic splice isoforms are involved in various cellular processes, such as apoptosis, cell signaling and proliferation [11].

Strategies to inhibit the expression of oncogenes and the carcinogenic splice variants of essential genes at the mRNA level have become popular in the past few decades. Pre-mRNA or mRNA with known nucleotide sequences offer a chance to design antisense oligonucleotides (AOs) specific to the transcripts. AOs are short 15–30-base long DNA or RNA molecules that are chemically modified [12]. AOs are designed to be complementary to the target sequence. AOs bind to the target RNA through Watson–Crick base pairing. The specificity of AOs depends on the accuracy of the Watson–Crick base pairing on the estimate that a particular sequence of >16 bases occurs only once in the human genome [13]. Typically, AOs are chemically modified to improve target binding affinity and stability [14,15]. Efficient delivery of AOs into target cells is challenging. Strategies for the delivery of AOs are continually improved to ease the entry of the AOs into the cells [16].

**Figure 1:**
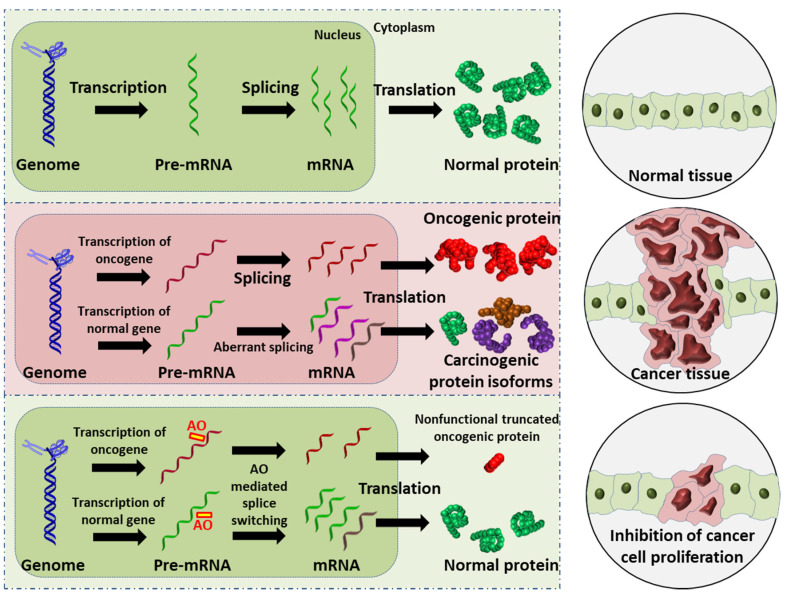
Schematic illustration of the AO-induced anticancer effect. (**Upper**): normal cell and tissue. (**Middle**): activation of oncogene leads to production of oncogenic proteins, and the aberrant splicing of normal gene results in splice variants that are translated into carcinogenic protein isoforms; these carcinogenic proteins collectively promote cancer cell proliferation. (**Lower**): AO-mediated splice switching can not only correct the aberrant splicing, thus reducing the production of carcinogenic protein isoforms (this is the focus of the present review), but can also induce premature stop codons in oncogenic mRNA that results in the synthesis of largely truncated, nonfunctional oncogenic proteins (this is a potential anticancer strategy). Collectively, AO intervention can inhibit cancer cell proliferation.

Mechanisms of action of AOs are elucidated in the next section. AOs that induce RNase H-mediated mRNA degradation have been widely used for downregulation of oncogenes [11,17,18]; therefore, this review focuses primarily on six distinct studies that use AO-mediated splice modulation as a therapeutic strategy, with a discussion of different chemical modifications used and delivery strategies employed. In addition, although premature termination of a codon induced by AO-mediated exon skipping has not yet been reported for the development of anticancer AOs, this can potentially be a useful strategy of AO-based cancer therapy (Figure 1).

## 2. Mechanism of Action of AOs

**Figure 2:**
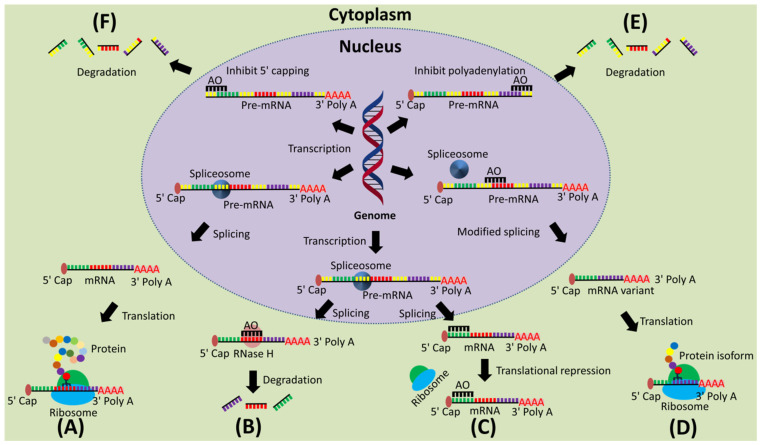
Mechanism of action of AOs to alter gene expression. (**A**) Normal gene expression process; (**B**) AO-induced RNase H-mediated mRNA degradation; (**C**) AO-induced translational repression; (**D**) AO-induced splice modulation; (**E**) AO-induced inhibition of polyadenylation; (**F**) AO-induced inhibition of 5′ capping.

A multitude of proteins are responsible for the normal functioning of cells. However, proteins are coded by a smaller number of genes. Briefly, alternative splicing is a process where the same gene can code for multiple proteins via exon inclusion, exon skipping or trimming of specific exons (Figure 2A) [17]. Aberrant splicing can be a cause of many disorders, including cancer. Correction of aberrant splicing using AOs is a widely studied approach. AOs are chemically modified short oligonucleotide that targets a specific mRNA or pre-mRNA by hybridization through Watson–Crick base pairing. These oligonucleotides inhibit the function of the RNA. Typically, there are three different mechanisms that are employed in the modulation of RNA function. They are (i) RNase-H mediated cleavage of the mRNA-DNA duplex (Figure 2B), (ii) steric block-mediated translation arrest (Figure 2C,E,F), and (iii) modulation of the pre-mRNA splicing to regulate expression of the target gene (Figure 2D) [12,17].

RNase H-mediated cleavage is activated when an AO binds to a complementary target mRNA, forming a DNA-mRNA heteroduplex. The duplex recruits the ubiquitously expressed RNase-H endonuclease enzyme that selectively cleaves the duplex by hydrolyzing the inter-nucleotide phosphate linkage and thereby inhibiting target gene expression [19]. This approach is particularly effective when the AOs are made of oligodeoxyribonucleotides. On the other hand, a steric block involves the AOs physically blocking the binding motifs of interacting proteins, such as at the 5′-cap site, ribosome binding site or translation start site [20].

**Figure 3:**
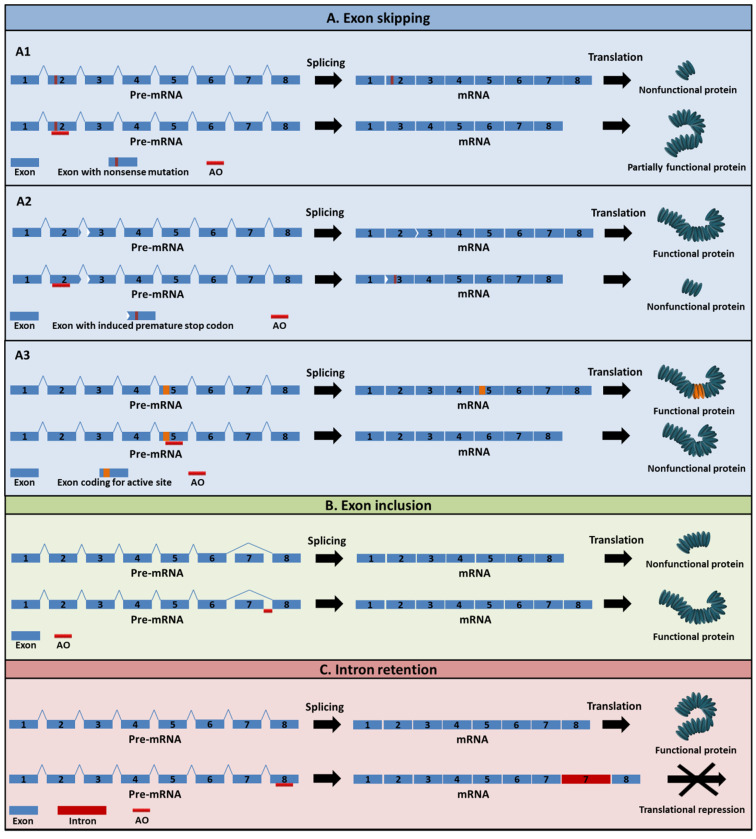
Representation of AO-mediated splice modulation, including (**A**) exon skipping, (**B**) exon inclusion, and (**C**) intron retention. Specially, exon skipping can be used to (**A1**) remove nonsense mutation to restore essential protein expression, or (**A2**) induce a premature stop codon or (**A3**) remove an exon-encoding active site to downregulate the expression of an unwanted protein. Exon inclusion can be used to increase the production of an essential protein, while intron retention may be used to reduce the expression of an unwanted protein by inducing translational repression.

AOs can also be designed to target pre-mRNA to modulate RNA processing. Splice-switching AOs can manipulate processes such as exon skipping, exon inclusion and intron retention (Figure 3) by targeting the pre-mRNA splicing factor binding sites. AOs binding to the target mRNA can effectively alter the reading frame of the transcripts by inducing exon skipping, allowing the production of a partially functional protein [21]. This attribute of AOs has been well studied in the treatment of DMD [22]. In DMD, mutations in the exon leading to a premature stop codon is rectified by removing the mutated exon. Exondys 51 (eteplirsen) and Vyondys 53 (golodirsen) are two such FDA-approved drugs that target exon 51 and 53, respectively [23,24,25]. It is also observed that AO-mediated exon skipping can disrupt the reading frame and knock down the protein [26]. In theory, AOs can also be used to target exonic or intronic silencers and can restore an exon in a disease-associated exon skipping framework.

In addition, AOs can also target other sites in the mRNA, including cleavage stimulation factor (CstF), polyadenylation specificity factor (CPSF) or polyadenylation signal sequences, and can stop the process from promoting translation arrest [12,27]. Splice-modulating AOs can also be utilized to cause exon inclusion and intron retention in addition to exon skipping. The exon-inclusion strategy has worked well both in vitro and in vivo as a therapeutic approach for spinal muscular atrophy (SMA) [28]. Nusinersin (Spinraza), an approved drug for SMA, works by modulating the splicing of the *SMN2* gene, which facilitates the integration of exon 7 into the mRNA, thus enhancing full-length SMN protein production in the central nervous system [29]. Figure 3 summarizes the techniques employed by AOs with particular emphasis on exon-skipping AOs. Although AOs are diligent in inducing exon skipping for a robust therapeutic effect, it is essential that they reach the target region in the desired amount. To enhance the potential of AOs, various chemical modification and delivery mechanisms are employed.

## 3. Chemical Modifications

Antisense oligonucleotides made of natural nucleotide monomers are not suitable for therapeutic applications. Stability against nucleases, target-specific binding, tissue distribution and cellular uptake are some of the major concerns with AOs composed of natural nucleotide monomers [30]. Chemically modifying the AOs addresses some of these limitations. Chemical modifications can be introduced at the backbone, nucleobase and sugar moiety. Some of the most commonly used chemical modifications are discussed in this section.

**Figure 4:**
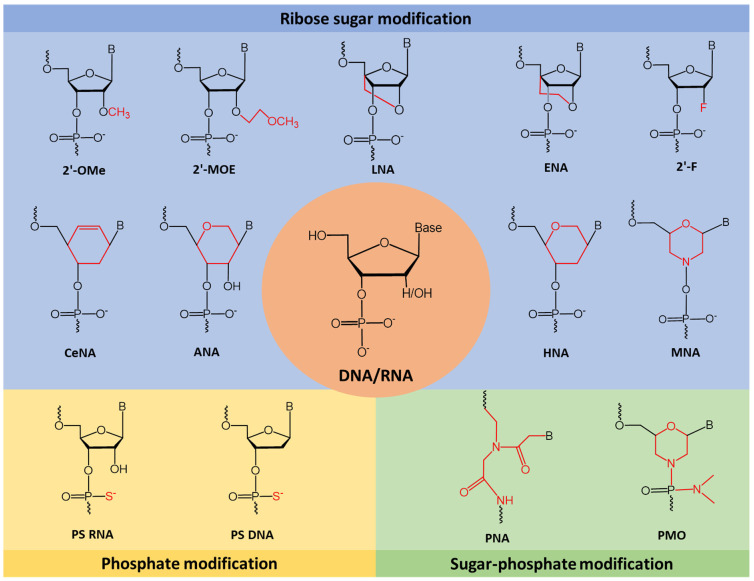
This figure depicts some of the most common or novel chemically modified nucleotide analogues used in constructing splice modulating AOs. 2′-OMe: 2′-O-methyl; 2′-MOE: 2′-O-methoxyethyl; LNA: locked nucleic acid; ENA: 2′-O, 4′-C-ethylene-bridged nucleic acid; 2′-F: 2′-fluoro; CeNA: cyclohexenyl nucleic acid; ANA: altritol nucleic acid; HNA: 1,5-anhydro hexitol nucleic acid; MNA: morpholino nucleic acid; PS: phosphorothioate; PNA: peptide nucleic acid; PMO: phosphorodiamidate morpholino oligomer.

Changes made to the phosphodiester backbone usually replace the non-bridging oxygen atom with carbon (methyl phosphonate, phosphotriester), sulphur (phosphorothioate), nitrogen (phosphoroamidate) or boron (boranophosphate) [31]. Modifying the backbone provides AO stability against nuclease degradation [11]. Examples of sugar modifications include 2′-O-methyl (2′-OMe), 2′-O-methoxyethyl (2′-MOE), locked nucleic acid (LNA), 2′-fluoro RNA (2′-F), hexitol nucleic acid (HNA), cyclohexenyl nucleic acid (CeNA), altritol nucleic acid (ANA), 2′-O, 4′-C-ethylene-bridged nucleic acid (ENA), and morpholino nucleic acid (MNA) (Figure 4). Alterations to the sugar moiety generally increase the binding affinity of AOs, protects AOs from nuclease degradation and increases its specificity [17].

Nucleobase modifications are not very common, as they impact the base-pairing properties of the AO. However, there is one naturally occurring nucleobase modification: the C-5 methyl substitution on pyrimidine nucleobases. This naturally occurring modification has been explored for its potential use in medicinal chemistry. Nucleobase modifications are not commonly seen in exon-skipping AOs. A detailed review of the various chemical modifications can be found elsewhere [32].

One of the first AOs to be used in exon-skipping experiments was 2′-OMe-PS [33]. These AOs have a phosphorothioate (PS) backbone with a 2′-O-methyl modified sugar moiety. The 2′-OMe-PS AOs were first used to show exon skipping in the dystrophin transcripts in cultured primary cells from *mdx* dystrophic mice [34]. Interestingly, Lu et al. demonstrated that 2′-OMe-PS AOs injected locally in skeletal muscles or via the tail vein showed efficient exon skipping and restored dystrophin protein levels [35,36]. This work led to the discovery of drisapersen, a 2′-OMe-PS AO drug targeting exon 51 that demonstrated significant restoration of the dystrophin protein and forbearance in systematic clinical trials [37].

Another chemical modification that has been studied is the use of tricyclo-DNA (tcDNA) to induce exon skipping in the treatment of DMD [38,39] or to induce exon inclusion for the treatment of SMA [40]. tcDNA deviates from natural DNA by the presence of three additional carbon atoms between c5′ and c3′, to which a cyclopropane unit is attached to further enhance structural stability [41,42]. tcDNA chemistry displays interesting properties for neuromuscular disorder therapy due to its quick uptake in cardiac and respiratory muscles [43]. This chemistry also has the unique property of being capable of crossing the blood-brain barrier at low levels after systemic uptake [38,39,40].

Locked nucleic acid (LNA)-modified AOs have a methylene bridge connecting 2′-O and 4′-C of the ribose sugar [44,45]. LNA AOs were shown to induce effective exon 46 skipping in exon 45-deleted DMD patient cells. LNA AOs showed effective skipping and exhibited high specificity due to the high target affinity of the 14-mer LNA AO [46]. Peptide nucleic acids (PNA) have an N-(2-aminoethyl)-glycine unit attached to the nucleobase and show good affinity to both DNA and RNA targets with high specificity [47]. In a DMD model system, PNA oligonucleotides, along with other PNA conjugates such as the trans activator of transcription protein (TAT protein), adeno-associated virus (AAV) 6 and AAV-8 functional domain and muscle-specific peptides were used to induce exon 23 skipping in vitro and in vivo. PNA AOs and PNA along with the conjugates effectively induced exon 23 skipping in both in vitro and in vivo models [48]. In another extended study, a 25-mer PNA targeting exon 23 showed better exon skipping than its 20-mer PNA counterpart [49].

Phosphorodiamidate morpholino oligomers (PMO) are made of DNA bases linked to a methylenemorpholine ring backbone attached through phosphorodiamidate groups. In general, PMOs are more effective in modulating splicing when compared with a corresponding 2′-OMe-PS AO [50]. In a direct comparison study in *mdx* mice between a 25-mer PMO and the same length 2′-OMe-PS AO, the PMOs induced better exon 23 skipping and showed higher levels of dystrophin protein recovery upon both systemic and local delivery than the corresponding 2′-OMe-PS AO [51]. In another comparison study, PMOs showed more exon 23 skipping in a mouse model than the 2′-OMe-PS AO [52]. However, in human exons, both PMO and 2′-OMe-PS AO indicated comparable data in vivo. In addition, PMOs might be less sequence-specific than 2′-OMe-PS AO [53]. More recently, the use of peptide-conjugated PMOs has emerged to facilitate better delivery of PMOs. Betts et al. showed that an arginine-rich cell-penetrating peptide conjugate effectively delivered PMOs to non-skeletal muscles. The delivery was quite efficient, as it increased heart activity and high dystrophin restoration. Their data also established that the PNA internalization peptides (Pip) sequence was efficient for PMO delivery [24,53]. In 2016, Exondys 51, a PMO drug for DMD developed by Sarepta Therapeutics, was FDA approved [54]. In 2019, another PMO drug, Vyondys 53, was also approved by the FDA, which was also developed by Sarepta Therapeutics [25]. The potential of various chemically modified oligonucleotide chimeras has been explored in recent years to further improve the AO designs for enhanced splice switching [55,56,57,58,59,60,61,62,63,64].

## 4. Alternative Splicing in Cancer

Cancer is one of the leading causes of death worldwide. The traditional therapies for cancer such as chemotherapy, radiotherapy and surgery fail to address the fundamental molecular pathologies associated with the development of cancer and thus lead to inadequate treatment and cancer recurrence. One of the most recent advancements in the cancer treatment regimen is the development of targeted therapies. Evaluating the roles for cancer-related genes in the development of a tumor is a progressing field and has provided a number of viable targets for antisense therapy [65]. Specifically, in cancer, cell signaling pathways represent multiple targets. The most promising targets are the ones closely involved in key cancer-meditating processes, such as apoptosis, cell proliferation, angiogenesis and metastasis [65].

Most cancers are genetic diseases which are caused mainly due to aberrant changes in two main types of genes, i.e., proto-oncogenes and tumor suppressors [66]. Aberrant splicing of a proto-oncogene or tumor suppressor could impact a regulatory mechanism, resulting in cancer as opposed to the normal functioning of the proteins [10]. For example, cyclin D-binding Myb-like protein 1 (*DMP1*) is a tumor suppressor gene in breast cancer [67]. DMP1 splicing results in DMP1α, DMP1β and DMP1γ [68]. DMP1α acts as tumor suppressor by transcriptionally upregulating ADP ribosylation factor (*ARF*), initiating anti-tumorigenesis in a tumor protein p53 (*P53*)-dependent manner and causing apoptosis [69]. On the other hand, DMP1β has a proliferative oncogenic response that is P53-independent. DMP1β is overexpressed in around 60% of breast cancers with poor prognosis [68,69].

In addition to oncogenes and tumor suppressors, alternative splicing of normal genes or splicing regulators plays an important role in cancer. This alternative splicing either promotes or suppresses cancer growth depending on the isoform expressed [70]. The following examples of alternative splicing promote cancer when one isoform is expressed and impede cancer when the other isoform is expressed. Some of the genes that play a role in proliferation due to alternative splicing include ribosomal protein S6 kinase beta-1 (*RPS6KB1*), MAP kinase-interacting serine/threonine-protein kinase 2 (*MKNK2*), fibroblast growth factor receptor (*FGFR*), cyclin D1 (*CCND1*) and *H-RAS*. Aberrant splicing of BCL2-like 1 (*BCL2L1*), myeloid cell leukemia sequence 1 (*MCL-1*), fas cell surface death receptor (*FAS*), bridging integrator 1 (*BIN1*) and caspase 2 (*CASP2*) plays a role in cell death. Cancer cell metabolism is modified by alternative splicing of pyruvate kinase M1/2 (*PKM*) and bromodomain-containing 4 (*BRD4*) genes. Angiogenesis in cancer can be modulated by alternative splicing of vascular endothelial growth factor A (*VEGFA*). Specific isoforms of *CD44*, ENAH actin regulator (*ENAH*), Kruppel-like factor 6 (*KLF6*), recepteur d’Origine nantais (RON) tyrosine kinase, and Rac family small GTPase 1 (*RAC1*) can promote cancer cell invasion and migration. In addition to these, isoforms of BIM and HER2 impart drug resistance in cancer cells [30,71,72,73,74].

## 5. Exon-Skipping AOs in Cancer

The development of nucleic acid-based therapeutics for cancer has gained prevalence in the past decades. Splice-modulating AOs have been used for suppressing the genes involved in the progression of cancer [75,76]. In this section the existing exon-skipping antisense oligonucleotide studies are discussed.

### 5.1. Breast Cancer

In 2018, there were about 2.1 million newly diagnosed breast cancer cases in females worldwide, accounting for about one in four cancer cases in women. Only 5–10% of breast cancers are attributed to hereditary factors [77]. In general, breast cancers are categorized based on the expression of estrogen receptor, progesterone receptor and the human epidermal growth factor receptor 2 (HER2). Knowing the receptor status of cancer aids in providing specific treatment options. Approximately 25–30% of breast cancers have HER2 overexpression or amplification. Although conventional anti-HER2 therapy has achieved success in treating the HER2+ve subtype of breast cancer, the non-specific action of the HER2 inhibitors has limited the practicality of this approach [78,79,80]. So far, there are three studies elucidating the use of AO-mediated exon skipping in breast cancer. These studies mainly focus on the genes encoding epidermal growth factor receptors (*EGFR*), specifically *HER2* and human epidermal growth factor receptor 4 (*HER4*).

A 2′-MOE-modified AO was used to induce skipping of exon 15 in the *HER2* gene in SK-BR-3 breast cancer cells, leading to downregulation of HER2. Downregulation of HER2 results in upregulation of Δ15HER2, which is an inhibitor of HER2 (Table 1, No. 1). Δ15HER2 downregulated the full-length HER2 protein and reduced the transphosphorylation of HER3. These AOs served as a platform for the identification of the physiological role of Δ15HER2, a novel HER2 variant. In addition, administration of the AO inhibited the proliferation of the SK-BR-3 cells and induced apoptosis. This suggests that the 2′-MOE AO could be explored in the context of therapeutics [81]. In another in vitro study, a PNA targeting the intron-exon junction site of exon 19 of HER2 induced exon skipping in SK-BR-3 breast cancer cells and HeLa cervical cancer cell lines (Table 1, No. 2). Exon 19 codes for the ATP catalytic domain of HER2, leading to a functionally inactive HER2 protein in a dominant negative fashion. The PNAs used in this study could be a potential drugs for therapeutic applications [82]. While current studies have investigated HER2 in a breast cancer context only, the *HER2* oncogene is overexpressed in a variety of additional cancers as well, making a new drug widely applicable [83].

HER4, another member of the EGFR family, plays a diverse role in the development and progression of breast cancer [84]. LNAs targeting the intron-exon junction of exon 26 caused skipping of the exon and switched the splicing of HER4 from cytoplasmic domain 1 (CYT1) isoform to cytoplasmic domain 2 (CYT2) expression in HER4-expressing breast cancer cells (Table 1, No. 3). These AOs also inhibited the growth of the cancer cells. A similar splice switching pattern was observed in vivo in a xenografted tumor model subcutaneously injected with MCF-7 cells. Furthermore, the AO treatment also reduced tumor growth [85]. This shows that the use of LNAs targeting exon 26 of HER4 could be explored as a therapeutic strategy.

### 5.2. Leukemia

As of 2018, the incidence rate of leukemia globally is about 2.4% and the mortality rate about 3.2% [86]. Treatment options for leukemia vary depending on the type. Apart from conventional treatments like chemotherapy, the use of monoclonal antibodies and gene inhibitors for acute lymphocytic and acute myeloid leukemia are prevalent [87,88].

Wilms tumor gene (WT1) overexpression is generally a characteristic of acute leukemias and is associated with poor prognosis [89]. WT1 is located on 11p13 and encodes a zinc finger motif-containing transcription factor. This transcription factor is involved in the regulation of differentiation and growth [90]. Expression pattern analysis revealed that WT1 is involved in the early stage of hematological cell differentiation [90]. An AO targeting exon 5 of WT1 showed an upregulation of thrombospondin, a target gene of WT1 in HL-60 cells (Table 1, No. 4). Upregulation of thrombospondin correlated with cell death in HL-60 cells. K562 cells were also sensitive to the WT1 AOs [90,91]. WT1 is not only overexpressed in leukemia, but also in a variety of other cancers, including breast cancer, prostate cancer and embryonal cancers [92]. This suggests exploring the therapeutic benefits of these AOs in leukemia and other cancers.

Terminal deoxynucleotidyl transferase (TdT) is an intracellular protein which is expressed by lymphocytes in the bone marrow and thymus [93]. High levels of TdT are characteristics of certain lymphoblastic leukemias. TdT is now a well-established diagnostic marker for acute lymphocytic leukaemia [93,94]. In an in vitro study, 16-mer PNAs were used to target the 5′ and 3′ junctions of intron 7 of TdT in Molt-4 acute lymphoblastic leukemia cells. The specificity of the AO was confirmed by comparing it to a sequence with two base mismatches. Reverse transcriptase PCR (RT-PCR) showed inclusion of intron 7 and subsequently skipping of exon 7 in the TdT gene, leading to significantly reduced TdT expression levels (Table 1, No. 5). Downregulation of TdT was accompanied with an increase in the rate of apoptosis [95].

### 5.3. Melanoma

The global maximum incidence rate and mortality rate of melanoma is about 4.2% and 1.4%, respectively. Although melanomas are not a leading cause of cancer deaths, the incidence rates of melanoma have significantly increased in the past 50 years [96]. While inactivation of p53 is a common event in most cancers, melanomas with TP53 mutations are very rare. However, the MDM4 regulator of p53 (MDM4) is highly upregulated in most melanomas and promotes the survival of melanoma cells by antagonizing the pro-apoptotic function of p53. This makes MDM4 a key therapeutic target for melanoma [97]. Overexpression of MDM4 is caused by inclusion of exon 6, which leads to the expression of a full length MDM4. MDM4 exon 6 acts as nonsense-mediated decay (NMD) “NMD switch” [98]. Though this alternative splicing event is regulated by a number of factors, it was noted that the oncoprotein serine- and arginine-rich splicing factor 3 (SRSF3) is the key initiator of exon 6 inclusion in MDM4 [98]. In this study, a morpholino AO flanking the exon-intron junction of exon 6 and overlapping the SRSF3 binding site inhibited cancer growth both in in vitro and in vivo melanoma models (Table 1, No. 6). It also sensitized the cells to MAPK-targeting therapeutics, which is a common treatment strategy for melanoma. Similar results were seen in lymphoma models as well. As MDM4 is overexpressed in additional types of cancer, this strategy may be applicable beyond melanoma [98].

## 6. Antisense Oligos in Clinical Trials

A comprehensive list of AOs in cancer clinical trials has been enumerated in Table 2.

### 6.1. Apatorsen/OGX-427

Apatorsen is a 20mer AO harboring a 2′-MOE-PS modification. This AO effectively reduced the expression of heat shock protein 27 (Hsp27), an adenosine-independent cytoprotective chaperone overexpressed in cancers. A dose escalation phase I trial of apatorsen carried out in patients with advanced cancers revealed that a maximum dose of 1000 mg can be tolerated [99]. This drug, in combination with other chemo drugs, improved the survival of patients with poor prognostic advanced cancers [100,101]. In another trial, apatorsen with chemotherapy did not improve the overall and progression-free survival of metastatic pancreatic cancer, non-squamous non-small cell lung cancer (NSCLC), castration-resistant prostate cancer (CRPC) and squamous cell lung cancer patients [102,103,104,105].

### 6.2. AZD4785

AZD4785 is a 2′-4′ constrained ethyl (cEt) modified AO that binds to the 3′ untranslated region (3′UTR) of Kirsten rat sarcoma virus (KRAS) mRNA, leading to RNase H-mediated degradation. This cEt modification enables AO uptake by cells without any transfection reagent [106]. A phase 1 study investigated the safety and efficacy of AZD4785 in KRAS-driven advanced solid tumors. The results of this study are published on the AstraZeneca website [107].

### 6.3. AZD5312/AR_Rx_

The 2.5 generation AO AZD5312 is a PS cEt AO that binds to androgen receptor (AR) mRNA. A phase I dose escalation study of AZD5312 revealed that 900 mg is the maximum tolerated dose in patients with androgen receptor-driven advanced tumors [108]. This study did not show a favorable reduction in AR expression. Another phase I/II study is currently ongoing wherein AZD5312 is administered in combination with enzalutamide in advanced prostate cancer patients [109].

### 6.4. AZD9150/ISIS 481464/ISIS-STAT3_Rx_/Danvatirsen

Danvatirsen is a 16mer, 3-10-3 modified gapmer containing ten PS-modified deoxynucleotides flanked by three cEt nucleotides. Danvatirsen regulates the expression of signal transducer and activator of transcription 3 (*STAT3*). Phase I studies of danvatirsen in lymphoma and other advanced malignancies as a monotherapy and in combination with chemotherapies revealed the safety and efficacy of this AO [110,111,112]. Currently, danvatirsen is under phase II trials in advanced tumors to evaluate the tolerability and efficacy of the combination of drugs.

### 6.5. BP1001

BP1001 is synthesized using DNAbilize technology and is a dioleoylphosphatidylcholine (DOPC) lipid-encapsulated P-ethoxy modified AO. BP1001 targets growth factor receptor-bound protein 2 (Grb2), which is involved in oncogenic tyrosine kinase signal transduction. A dose escalation and expansion phase I clinical trial in advanced hematological cancers showed that BP1001 is well tolerated at 90 mg/m² concentration [113]. Phase II trials are currently recruiting acute myeloid leukemia (AML) patients to test the efficacy of BP1001 in combination with chemotherapeutic drugs such as venetoclax and decitabine.

### 6.6. C-Myb AS ODN/G4460/LR3001

The master regulator transcription factor c-Myb plays a major role in hematopoietic malignancies [114]. G4460 is a PS-modified 24mer AO that binds to codons 2 to 9 of c-Myb mRNA [115]. A phase II study was carried out in chronic myelogenous leukemia (CML) patients to study the efficacy of G4460 in combination with chemotherapy followed by bone marrow transplantation [116]. A phase I trial of c-Myb AO administered G4460 as a continuous intravenous infusion to patients with advanced hematological malignancies. The efficacy of c-Myb AO could not be concluded from these studies.

### 6.7. EZN-2968/RO7070179/SPC2968

The LNA-modified EZN-2968 is a 16mer AO that is complementary to the α subunit of hypoxia-inducible factor-1 alpha (HIF-1α). This third-generation AO is modified with LNA at bases 1 to 3 and 13 to 15, and DNA at 4 to 12 and 16 [117]. This AO entered phase I clinical trials to study its maximum dose and safety in patients with advanced cancers. EZN-2968 was well tolerated at a maximum dose of 4.1 mg/kg/day with no dose-limiting toxicities [118]. Another phase I trial carried out in patients with refractory tumors revealed reduced levels of HIF-1α post treatment [119]. The efficacy of EZN-2968 was tested in a phase I trial with hepatocellular carcinoma (HCC) patients unresponsive for systemic therapy. This study revealed that 10 mg/kg EZN-2968 was well tolerated in HCC patients with either stable disease or partial responses [120]. However, these studies need to be validated in more patients to estimate the effectiveness of this AO.

### 6.8. G3139/Oblimersen/Genasense

Oblimersen is a 18mer PS AO that binds to the first six codons of B cell lymphoma 2 (Bcl2) mRNA. It is tested as a monotherapy or in combinations to study its safety and efficacy in different cancer types. The first Phase I/II clinical trial of oblimersen was started in 1997 as a combination therapy with docetaxel in advanced solid tumors [121]. The next phase I study was carried out in refractory acute myeloid/lymphoblastic leukemia patients alongside fludarabine, cytarabine and filgrastim, which revealed that oblimersen is effective in combination with chemotherapy [122]. The following year, oblimersen combination therapy testing was initiated in phase I and II clinical trials in small cell lung cancer (SCLC), AML, and colorectal cancer (CRC) [123,124,125] and in phase III trials for malignant melanoma and multiple myeloma [126,127]. These studies showed that the combination therapies for respective cancers along with oblimersen were well tolerated and the adverse events of the combinations were similar to chemotherapy alone. The phase III trial with oblimersen in advanced multiple myeloma patients did not have any significant improvement in time to tumor progression when compared to dexamethasone treatment alone [127].

A phase I study of oblimersen combination therapy in extensive stage SCLC revealed that an oblimersen and chemotherapy combination had a better response rate and time to tumor progression [128]. A phase I/II trial in chronic lymphocytic leukemia (CLL) patients with oblimersen monotherapy was reported to have modest activity [129], and hence, a phase III trial was carried out in combination with fludarabine/cyclophosphamide. A five-year follow-up of this phase III trial showed improved survival in 45% of responding patients and 50% reduced death in oblimersen combination treatment [130]. A phase I and II trial of oblimersen with chemotherapy showed improved outcomes, which led to a phase III trial in AML. Follow-up studies showed no significant difference in disease-free survival in patients treated with oblimersen in addition to chemotherapy [131]. A similar outcome was seen in a phase III trial of oblimersen and dacarbazine in advanced melanoma [132]. Even though there were some improvements in the oblimersen treatment, it did not improve the overall progression or disease-free survival of these cancer patients.

### 6.9. GTI-2040

GTI-2040 is a 20mer PS AO that binds to the R2 subunit of ribonucleotide reductase (RNR). A dose escalation phase I study of GTI-2040 in patients with advanced cancers revealed that 185 mg/m^2^/day is well tolerated as a monotherapy [133]. A phase I/II trial of GTI-2040 with capecitabine in advanced renal cell carcinoma (RCC) patients was not successful, and hence, it was not recommended to proceed further in RCC [134]. Phase I/II studies of GTI-2040 in combination with chemotherapies were carried out in different cancers, including metastatic breast cancer, AML, NSCLC, metastatic solid tumors and CRPC [135,136,137,138,139]. From these clinical trials, it was evident that GTI-2040 performed better as a single agent therapy than in combination with other drugs [140].

### 6.10. ISIS 3521

ISIS 3521 is a PS-modified 20mer oligo that is complementary to the 3′UTR of protein kinase C alpha (PKC-α). A dose escalation phase I study of ISIS 3521 in advanced cancer patients revealed 6 mg/kg as a recommended dosage [141]. A combination of ISIS 3521, gemcitabine and carboplatin/cisplatin was used in phase II/III clinical trials of advanced NSCLC patients. This study showed that the combination therapy did not improve the response rate or survival of advanced NSCLC patients [142].

### 6.11. ISIS 5132

Human c-Raf kinase targeting ISIS 5132 is a first-generation PS-modified AO with a length of 20 bases. The maximum tolerated dose was observed to be 4 mg/kg/day during a phase I trial in advanced cancer patients [143]. A phase II trial of ISIS 5132 in advanced ovarian cancer patients showed no clinical activity [144]. These trials concluded that ISIS 5132 cannot be used as a single agent therapy. A phase II study of a combination of ISIS 3521 and ISIS 5132 was carried out in metastatic CRC patients. This study also did not show any positive clinical outcome [145].

### 6.12. LErafAON

LErafAON is a c-Raf-1-targeting AO ISIS 5132 encapsulated in a cationic liposome; it is about 400 nm diameter. A phase I trial of LErafAON in advanced solid tumors resulted in dose-dependent and dose-independent adverse events [146]. Another phase I trial was carried out in advanced cancer patients using a combination of LErafAON alongside radiotherapy. This study reported similar adverse events as the previous study and hence concluded that the liposomal preparations need to be modified [147].

### 6.13. OGX-011

Custirsen/OGX-011 is a second-generation 2′-MOE PS-modified AO that is complementary to clusterin mRNA. A phase I dose escalation study of OGX-011 performed in prostate cancer patients discovered that 640 mg was the optimal dosage [148]. Following this study, a combination therapy phase I clinical trial was carried out in patients with advanced cancer. It was observed that overall toxicity was moderate with increased gastrointestinal effects with an increase in OGX-011 concentration [149]. In a phase II clinical trial with metastatic breast cancer patients, an OGX-011 and docetaxel combination was well tolerated, and it did not have any adverse events other than the docetaxel-mediated ones. Additionally, there was not much difference in the response rate between the mono- and combination therapy [150]. A phase II study in castration-resistant prostate cancer patients with OGX-011 in combination with chemotherapy showed improved clinical outcomes [151,152]. A large phase III clinical trial of OGX-011 and chemo combination therapy in castration-resistant prostate cancer patients did not show any improved outcomes relevant to OGX-011 [153].

## 7. Modes of AO Delivery

The effectiveness of an AO depends primarily on the extent to which the AO diffuses through cells. AOs require an appropriate delivery system to facilitate their entry into the cytoplasm and/or the nucleus. One of the key issues faced is the process of endosomal escape. Delivery systems employed for AOs are constantly being updated to ensure optimal delivery of the AOs [186,187,188]. In this section, some of the delivery modes used, such as liposome- and lipoplex-based delivery systems, dendrimers, polymers, peptides, macrophages, electroporation, and light are discussed in brief.

Lipidic delivery systems are the most commonly used delivery vehicles in AO transport. Lipoplexes are a combination of cationic liposomes, neutral lipids and nucleic acids which ease the intracellular delivery of AOs [189]. The most widely used cationic lipids include DOTAP (N-[1- (2,3-dioleoyloxy) propyl]-N,N,N-trimethylammonium methylsulfate) and DOTMA (N-[l- (2,3-dioleyloxy) propyl]-N,N,N-triethylammonium) [190,191]. Lipoplexes enter cells through endocytosis. The neutral lipid dioelylphosphatidylethanolamine (DOPE) facilitates the release of the AOs through fusion and disruption of the endosomal complex [192]. A detailed review on lipid-based delivery vehicles can be found elsewhere [193]. There are many commercially available lipid-based delivery agents which have been studied extensively and have been shown to improve AO delivery [194].

Dendrimers are a supermolecular delivery system which can be synthesized with various functional groups, making them a versatile non-viral particle delivery system [195]. The commonly used dendrimer polyamidoamine (PAMAM)starburst possess a hydrocarbon core, charged amino groups and a controlled chemistry which is suitable for anionic AO delivery [195]. Belinshka et al. elucidated the use of dendrimers as a delivery system for AOs in cells that express luciferase. This study showed around 25–50% reduction of baseline luciferase activity, indicating the effectiveness of dendrimer-based delivery of AOs [196].

Polymers are also employed as delivery vehicles. Biodegradable polymers can offer sustained release of AOs. Poly-L-lysine is one of the first studied cationic polymers used for in vivo gene transfer [197]. Polylactides and copolymers of lactic acid and glycolic acid P(LA-GA) are the most commonly studied polymers; these polymers facilitate the delivery of AOs at a controlled pace and offer nuclease resistance [198,199]. P(LA-GA) has also shown effective transfection of AOs when compared with free AO uptake [200]. Hydrogels are hydrophilic polymers that form three-dimensional networks via chemical or physical crosslinks. Hydrogels mediate the sustained release of AOs in response to pH, concentration differences of analyte ionic strength electric fields or temperature [201]. A cationic hydrogel made of c-agarose coupled with AO against tumor necrosis factor alpha (TNF-α) showed high target affinity to the spleen and alleviated inflammation in arthritis animal models [202]. Lou et al. showed that a poly [1-vinyl-2-pyrrolidinone-co-(2-hydroxyethyl methacrylate)] hydrogel effectively delivered AOs to the specific target [203].

Peptide-based delivery vectors facilitate the delivery of AOs via endocytosis. Cell penetrating peptide (CPP) facilitates intracellular uptake unescorted [204]. TAT derived from HIV and penetratin from antennapedia homeodomain were the first CPPs used [205,206]. Amphiphilic anionic peptides like E5CA are able to effectively deliver AOs to the cytosol and nucleus [207]. CPPs have found applications in both in vitro and in vivo studies [208].

Novak et al. have elucidated a fairly new approach to deliver AOs via macrophages. Inflammatory cells work as a depot for the administered AOs, which are then released along with the damage repair protein and are readily available for use by the regenerating cells. This mode of AO delivery has been explained clearly in a DMD system, which improves site-specific delivery of the AO [209]. Exosomes are extracellular microvesicles which are about 40–100 nm in diameter [210]. Initially thought to be insignificant in cellular functioning, exosomes have now been identified to play major roles in systemic cell-cell communication [211,212]. In spite of their nanoscale, exosomes can encapsulate bioactive molecules in a single lipid membrane [213]. Exosomes are also highly cell type-specific and hence are perfect candidates for the specific delivery of nucleic acids. [214]. For example, an LNA-modified AO for the suppression of miR34a successfully inhibited chondrocyte growth in osteoarthritis treatment [215].

Electroporation uses an externally applied electric field to create pores in the cell membrane when facilitating the entry of the AOs into the cells. Electroporation is a localized mode of AO delivery to the cells. Zewert et al. and Reigner et al. showed that fluorescence-labelled AOs against specific targets entered cells effectively through electroporation [216,217]. In another study, electroporation of AOs against proto-oncogene c-myc yielded specific suppression of c-myc [218]. Reigner et al. also showed effective transdermal delivery of phosphorothioate AOs in vivo [219].

Light-triggered delivery of AOs is a new technique established in the past decade. Fluorescent labelled AOs are generally conjugated with protein VP22 obtained from herpes simplex virus. This AO-protein complex is known as a vectosome. This vectosome reaches the cytoplasm and can be triggered by light, which releases the AO and the protein complex [220,221,222]. In vivo intravitreal injection of the vectosomes followed by transscleral illumination allowed delivery of AOs to retinal and RPE cells [222]. Recently, light-mediated delivery was carried out by complexing AOs with a thermosensitive liposome that releases the AOs upon light exposure, which showed high transfection efficiency [223].

Ultrasound-mediated AO delivery is another physical method that uses ultrasound waves of an optimal frequency. The AOs are generally delivered into the system using microbubbles, which then burst open by a localized application of ultrasound waves [224,225]. Negishi et al. elucidated this approach in a DMD mouse model *(mdx)* where a PEG-modified bubble liposome with ultrasound contrast gas was used to deliver the specific PMO [226].

## 8. Conclusions

The studies elucidated in this review reveal that antisense-mediated splice-switching strategy can be effectively used to change the expression of oncogenes and thereby control tumor growth in cancer models. A major drawback in the development of antisense drugs is site-specific delivery and stability. With the advent of new delivery techniques and chemical modifications, this caveat can likely be overcome in the future. Recent studies have also shown that chemically modifying the AOs increased the stability and the binding affinity of the AOs. Furthermore, these AOs were more cost effective with reduced cytotoxicity. This has paved the way for more targets to be explored that underpin a plethora of diseases, taking a step further in finding a possible cure. Antisense technology has progressed rapidly in the past two decades and has now emerged as a highly explored therapeutic strategy that is less or non-toxic and more efficacious.

## Figures and Tables

**Table 1:** Schematic representation of the six distinct studies on developing splice-modulating AOs targeting cancers. Among these AOs, (**1**) SSO111, (**2**) Acr-PNA 2794, (3) SSOe26, and (**6**) morpholino MDM4 inhibited overexpression of oncogenes by inducing exon skipping, thus producing non-functional variants or leading to nonsense-mediated decay (NMD) of mRNA, while (**5**) PNA 4577, 4578, 4580 and 4581 predominantly induced intron retention, resulting in the production of non-functional variants. Besides, (**4**) ASWT1exon5 induced RNase H-mediated degradation of longer transcripts, thus increasing the proportion of naturally occurring, shorter transcripts which exclude an important exon. 2′-MOE, 2′-O-methoxyethyl; 2′-OMe, 2′-O-methyl; PNA, peptide nucleic acid; LNA, locked nucleic acid; PS, phosphorothioate; PMO, phosphorodiamidate morpholino oligomer.

No.	Research		Ref.
1	AO	SSO111 is a 20mer fully modified 2′-MOE-PS AO-targeting oncogene *HER2*. SSO111 induced exon 15 skipping during splicing, leading to the generation of a novel mRNA transcript that excludes exon 15. 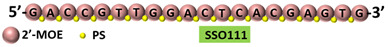	[81]
	Mechanism	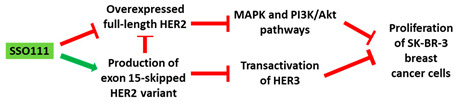	
2	AO	Acr-PNA 2794 is a 15mer fully modified PNA AO conjugated with Acr targeting *HER2*. Acr-PNA 2794 induced exon-19 skipping, leading to the generation of a novel mRNA transcript that excludes exon-19. 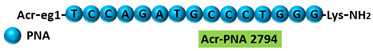	[82]
	Mechanism	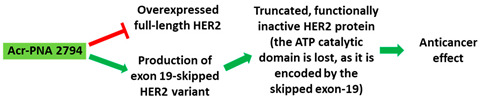	
3	AO	SSOe26 is a 15mer LNA-modified mixmer AO targeting *HER4*. SSOe26 induced exon 26 skipping, leading to the generation of a novel mRNA transcript that excludes exon 26 (CYT2 isoform). 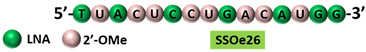	[85]
	Mechanism	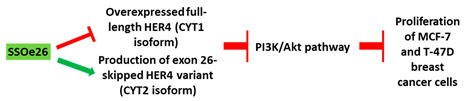	
4	AO	ASWT1exon5 is a 20mer 2′-MOE-PS gapmer AO targeting oncogene *WT1*. It induces RNase H-mediated degradation of exon 5-containing transcripts, thus increasing the proportion of transcripts that exclude exon 5. 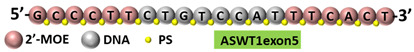	[91]
	Mechanism	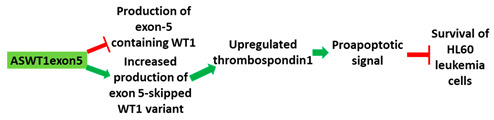	
5	AO	PNA 4577, 4578, 4580, and 4581 are 16mer fully modified PNA AOs conjugated with octaarginine or cholic acid-targeting oncogene *TdT*. These four PNAs all induced intron 7 retention, leading to the generation of a novel mRNA transcript that included intron-7. 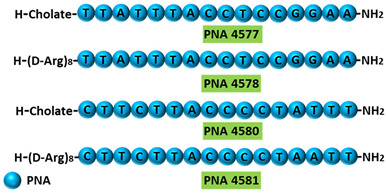	[95]
	Mechanism	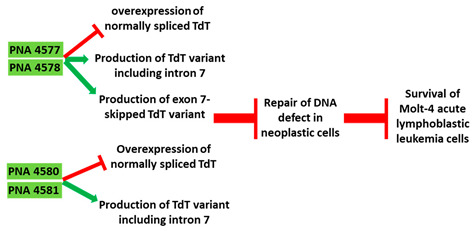	
6	AO	Morpholino MDM4 is a 25mer fully modified PMO AO targeting *MDM4*. Morpholino MDM4 induced exon 6 skipping, leading to nonsense-mediated decay of the mRNA transcript that excludes exon-6. 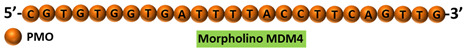	[98]
	Mechanism	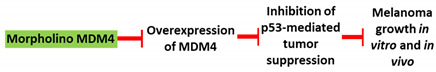	

**Table 2:** List of antisense oligonucleotides in clinical trials.

No	Drug Name	Target	Condition	Clinical Trial Number	Development Stage	Chemistry	Ref.
1	Apatorsen (OGX-427)	Hsp27	Prostate, ovarian, NSCLC, breast or bladder cancer	NCT00487786	Phase I	2′-MOE PS	[99]
			Prostate cancer	NCT01120470	Phase II		[104]
				NCT01681433	Phase II		[154]
			Urologic neoplasms	NCT01454089	Phase II		[100]
			Urothelial carcinoma	NCT01780545	Phase II		[101]
			Non-squamous NSCLC	NCT01829113	Phase II		[105]
			Advanced squamous cell lung cancers	NCT02423590			[103]
			Pancreatic cancer	NCT01844817	Phase II		[102]
2	AZD4785	KRAS	NSCLC/advanced solid tumors	NCT03101839	Phase I	2′-4′ cEt	[107]
3	AZD5312 (ARRx)	Androgen receptor	Androgen receptor-dependent advanced solid tumors	NCT02144051	Phase I	PS cEt	[108]
			Prostate cancer/solid tumors	NCT03300505	Phase Ib/II (recruiting)		[109]
4	AZD9150	STAT3	DLBCL/NHL	NCT01563302	Phase I/II	cEt gapmer	[110]
				NCT03527147	Phase I		[155]
			HCC and metastasis	NCT01839604	Phase I		[156]
			Advanced solid malignancies	NCT03394144	Phase I		[157]
			DLBCL	NCT02549651	Phase Ib		[112]
			Advanced solid tumors	NCT03421353	Phase I		Ongoing
			Muscle-invasive bladder cancer	NCT02546661	Phase I		Ongoing
			Metastatic NSCLC	NCT03819465	Phase I		Ongoing
			NSCLC	NCT03334617	Phase II		Ongoing
			Advanced solid tumors and metastatic squamous cell carcinoma of the head and neck	NCT02499328	Phase II		Ongoing
			Advanced pancreatic, NSCLC, and mismatch repair-deficient CRC	NCT02983578	Phase II		Ongoing
5	BP1001	Grb2	Recurrent adult AML/ALL Myelodysplastic syndrome Ph1-positive CML	NCT01159028	Phase I	DNAbilize^®^ technology	[113]
			Solid tumors	NCT04196257	Phase I		Ongoing
			AML	NCT02781883	Phase II		Ongoing
6	c-myb AS ODN	c-myb	Hematologic malignancies	NCT00780052	Phase I	PS	[158]
			Leukemia	NCT00002592	Phase II		[116]
7	EZN-2968	HIF-1	Carcinoma/lymphoma	NCT00466583	Phase I	LNA	[118]
			Liver metastases/neoplasms	NCT01120288	Phase I		[119]
			HCC	NCT02564614	Phase I		[120]
8	G3139 (Oblimersen)	Bcl-2	Waldenström Macroglobulinemia	NCT00062244	Phase 1 Phase 2	PS	[159]
			Merkel cell carcinoma	NCT00079131	Phase 2		[160]
			Solid tumors	NCT00003103	Phase 1 Phase 2		[121]
				NCT00054548	Phase 1		[161]
				NCT00543231	Phase 1		
				NCT00636545	Phase 1		[161]
			Relapsed or refractory solid tumors	NCT00039481	Phase 1		[162]
			Leukemia (AML or ALL)	NCT00004862	Phase 1		[122]
			AML	NCT00039117	Phase 1		[163]
				NCT00017589	Phase 2		[125]
				NCT00085124	Phase 3		[131]
			CRC	NCT00004870	Phase 1 Phase 2		[124]
				NCT00055822	Phase 1 Phase 2		[164]
			Prostate cancer	NCT00085228	Phase 2		[165]
			HCC	NCT00047229	Phase 2		[166]
			Recurrent SCLC	NCT00005032	Phase 1 Phase 2		[123]
			Extensive stage SCLC	NCT00017251	Phase 1		[128]
				NCT00042978	Phase 2		[128]
			CML	NCT00049192	Phase 2		[167]
			NHL	NCT00086944	Phase 1 Phase 2		[168]
			Recurrent B-cell NHL	NCT00054639	Phase 2		[168]
			CLL	NCT00078234	Phase 1 Phase 2		[169]
				NCT00021749	Phase 1 Phase 2		[129]
				NCT00024440	Phase 3		[130]
			Metastatic RCC	NCT00059813	Phase 2		[170]
			Advanced esophageal, gastro-esophageal junction and gastric cancer	NCT00064259	Phase 1 Phase 2		[171]
			Melanoma	NCT00409383	Phase 1		Unknown
				NCT00542893	Phase 1		[172]
				NCT00518895	Phase 3		[173]
				NCT00016263	Phase 3		[126]
				NCT00070343	Not applicable		Ongoing
			NSCLC	NCT00030641	Phase 2 Phase 3		Ongoing
			Multiple myeloma and plasma cell neoplasm	NCT00049374	Phase 2		[174]
				NCT00017602	Phase 3		[127]
			DLBCL	NCT00070083	Phase 1		
9	GTI-2040	R2 component of RNR	Breast cancer	NCT00068588	Phase 2	PS	[135]
			AML	NCT00070551	Phase 1		[175]
				NCT00565058	Phase 2		[136]
			Acute leukemia, high-grade myelodysplastic syndromes, or refractory or blastic phase CML	NCT00459212	Phase 1		[176]
			RCC	NCT00056173	Phase 1 Phase 2		[134]
			NSCLC, prostate cancer, or other solid tumors	NCT00074022	Phase 1 Phase 2		[137]
			CRC or other solid tumors	NCT00084643	Phase 1		[138]
			Prostate cancer	NCT00087165	Phase 2		[139]
			Metastatic or unresectable solid tumors	NCT00078962	Phase 1		[177]
10	ISIS 3521	Pkc-Alpha	NSCLC/ melanoma (skin)	NCT00003989	Phase II	PS	
			NSCLC	NCT00017407	Phase III		
				NCT00042679	Phase II		[178]
				NCT00034268	Phase III		[142]
11	ISIS 5132	C-raf	Ovarian cancer	NCT00003892	Phase II	PS	[144]
12	ISIS 3521 + ISIS 5132	Pkc-Alpha C-raf	Breast cancer	NCT00003236	Phase II		[179]
13	LErafAON	Raf-1	Advanced solid tumors	NCT00024661	Phase I	Liposome encapsulated PS	[146]
				NCT00100672	Phase I		[180]
				NCT00024648	Phase I		[147]
14	OGX-011	Clusterin	Cancer	NCT01497470	Phase I	2′-MOE PS	[181]
			NSCLC	NCT00138658	Phase I/II		[182]
				NCT01630733	Phase III		Ongoing
			Prostate cancer	NCT00054106	Phase I		[148]
				NCT00258388	Phase II		[151]
				NCT00138918	Phase II		[181]
				NCT00327340	Phase II		[152,183]
				NCT01578655	Phase III		[153]
				NCT01188187	Phase III		[184,185]
			Breast cancer	NCT00258375	Phase II		[150]
			Solid tumors	NCT00471432	Phase I		[149]

Hepatocellular carcinoma—HCC; small cell lung cancer—SCLC; non-small cell lung cancer—NSCLC; diffuse large B cell lymphoma—DLBCL; non-Hodgkin’s lymphoma—NHL; colorectal cancer—CRC; acute myeloid leukemia—AML; acute lymphoblastic leukemia—ALL; chronic myelogenous leukemia—CML; chronic lymphocytic leukemia—CLL; renal cell carcinoma—RCC.
